# Combining Spatial-Temporal and Phylogenetic Analysis Approaches for Improved Understanding on Global H5N1 Transmission

**DOI:** 10.1371/journal.pone.0013575

**Published:** 2010-10-22

**Authors:** Lu Liang, Bing Xu, Yanlei Chen, Yang Liu, Wuchun Cao, Liqun Fang, Limin Feng, Michael F. Goodchild, Peng Gong

**Affiliations:** 1 State Key Laboratory of Remote Sensing Science, Jointly Sponsored by Institute of Remote Sensing Applications, Chinese Academy of Sciences, Beijing Normal University, Beijing, China; 2 Center for Earth System Science, Tsinghua University, Beijing, China; 3 Department of Geography, University of Utah, Salt Lake City, Utah, United States of America; 4 Department of Environmental Science and Engineering, Tsinghua University, Beijing, China; 5 Department of Environmental Science, Policy and Management, University of California, Berkeley, California, United States America; 6 Computational and Molecular Population Genetics, Institute of Ecology and Evolution, University of Bern, Bern, Switzerland; 7 State Key Laboratory of Pathogen and Biosecurity, Beijing Institute of Microbiology and Epidemiology, Beijing, China; 8 Key Laboratory for Biodiversity Science and Ecological Engineering, Ministry of Education, College of Life Science, Beijing Normal University, Beijing, China; 9 Department of Geography, University of California Santa Barbara, Santa Barbara, California, United States of America; Duke University Medical Center, United States of America

## Abstract

**Background:**

Since late 2003, the highly pathogenic influenza A H5N1 had initiated several outbreak waves that swept across the Eurasia and Africa continents. Getting prepared for reassortment or mutation of H5N1 viruses has become a global priority. Although the spreading mechanism of H5N1 has been studied from different perspectives, its main transmission agents and spread route problems remain unsolved.

**Methodology/Principal Findings:**

Based on a compilation of the time and location of global H5N1 outbreaks from November 2003 to December 2006, we report an interdisciplinary effort that combines the geospatial informatics approach with a bioinformatics approach to form an improved understanding on the transmission mechanisms of H5N1 virus. Through a spherical coordinate based analysis, which is not conventionally done in geographical analyses, we reveal obvious spatial and temporal clusters of global H5N1 cases on different scales, which we consider to be associated with two different transmission modes of H5N1 viruses. Then through an interdisciplinary study of both geographic and phylogenetic analysis, we obtain a H5N1 spreading route map. Our results provide insight on competing hypotheses as to which avian hosts are responsible for the spread of H5N1.

**Conclusions/Significance:**

We found that although South China and Southeast Asia may be the virus pool of avian flu, East Siberia may be the source of the H5N1 epidemic. The concentration of migratory birds from different places increases the possibility of gene mutation. Special attention should be paid to East Siberia, Middle Siberia and South China for improved surveillance of H5N1 viruses and monitoring of migratory birds.

## Introduction

Highly pathogenic avian influenza (HPAI) A subtype H5N1 virus was first detected in Guangdong, China in 1996. It has initiated several outbreak waves that swept across the Eurasia and Africa continents since 2003. The high fatality of over 60% in human infection has sparked a fear that a disastrous pandemic strain may arise by mutation or reassortment with currently circulating seasonal influenza virus [1]. Intervention efforts would be better made if the main transmission agents and their ways of transmission are better understood. However, our knowledge on the global spreading of H5N1 is still limited. There is an ongoing debate regarding how H5N1 is spreading around the world [2]–[5]. Long distance migration of wild birds, poultry production and trade are at the center of deliberation. Advancements in transportation, coupled with the global food chain, enable the virus to be spread anywhere. Migratory birds, especially wild waterfowl such as Anseriformes and Charadrillformes, are considered natural carriers of all avian influenza A viruses. However, what exactly the main agent of dispersal and the spreading mechanisms are remain undetermined. Most previous studies with evidences on the cause and spread of H5N1 were based on sporadic specific cases, such as virus detection in smuggling and illegal poultry trading [6], or isolation in wild bird populations [7], using virus isolation, sequencing and phylogenetic analyses. At the regional level, environmental and socio-economic risk factors favoring the reoccurrence of the viruses have been identified through statistical regression analysis [8]. However, a more comprehensive global analysis on the spreading mechanisms of H5N1 at various spatial scales has rarely been done [46].

**Figure 1:**
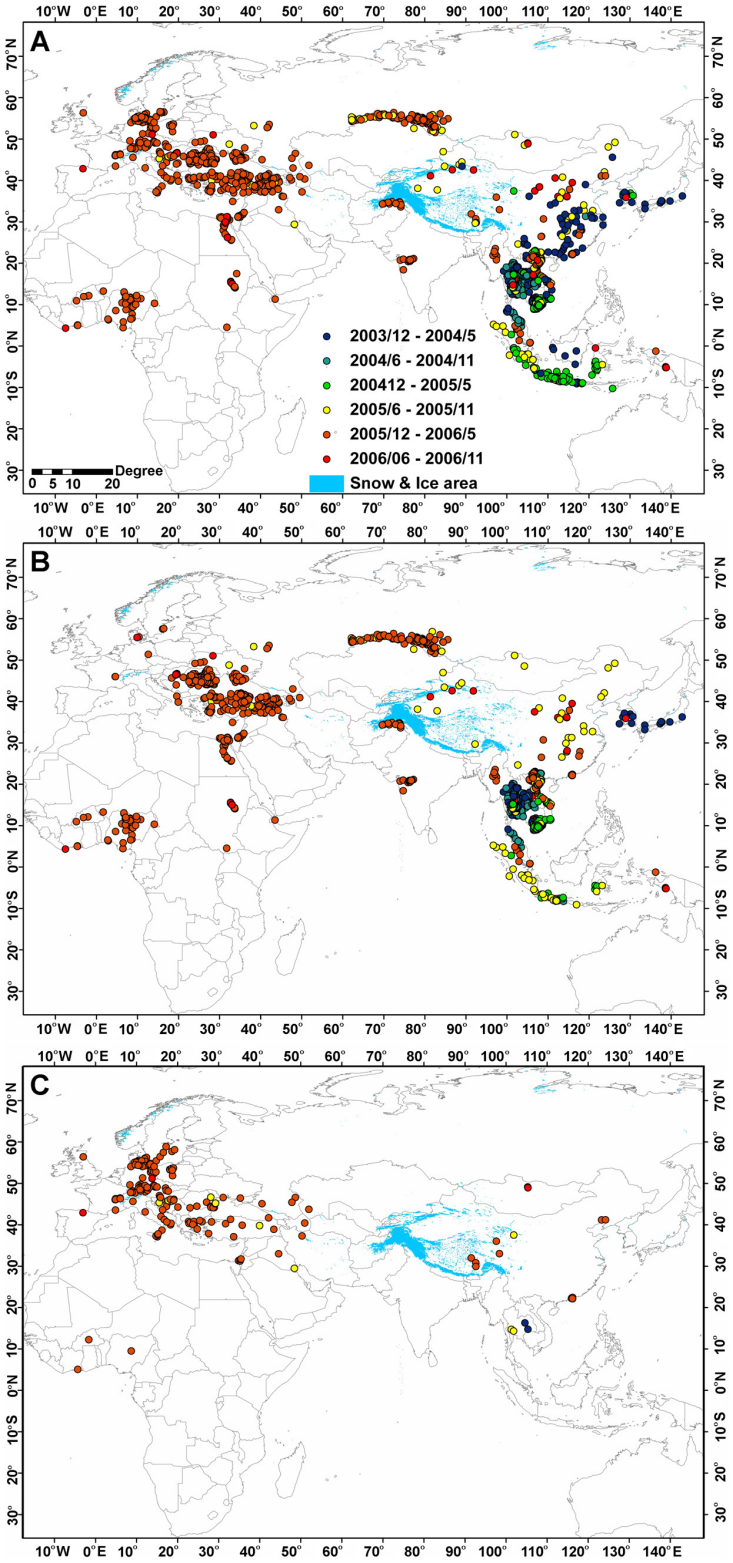
Distribution of global A) animal, B) poultry, C) wild bird H5N1 infections.

It is of interest to concentrate on how the virus is introduced into a new region. Once this issue is solved, it will be easier to implement surveillance and control strategies. Most previous studies make use of phylogenetic analysis to explore the emergency and circulation of a virus. Phylogenetic analysis is a powerful bioinformatics tool that can elucidate the phylogenetic relationships of virus isolates, give further insights into the origin of the virus, and provide clues to inferring putative pathways of introduction. But most phylogenetic analyses of H5N1 viruses were done at the regional to continental scales [9], [10]. It is insufficient to determine when, where, and how the virus is transmitting at the global scale. Furthermore, most analyses focused on the divergence and evolution of gene sequences, with little consideration on their geographic migration [11]. With a georeferenced global dataset [12], a study on the geographical analysis at the global scale is now possible [45]. Figure 1A shows the distribution of animal outbreak cases from November 2003 to December 2006, including 3,365 poultry and wild bird cases in 50 countries. In this study, we use 2,642 poultry cases (Figure 1B) and 334 wild bird cases (Figure 1C), while the remaining cases in the dataset are uncertain or incomplete.

In this paper we have three objectives: first, to determine the transmission mechanisms from the spatial temporal distribution of those global H5N1 outbreak cases; second, to delineate important introduction and spreading routes since 2003; and third, to identify the primary spreading agents for the long term and repeated circulation of the H5N1 virus.

## Results

### Spatial and Temporal Patterns of H5N1 Outbreak Cases

The global geographic H5N1 dataset registers each outbreak case at the location of the nearest village or township. We estimate most of the positional registration errors in densely populated areas to be within 10 km. A recent study estimates the maximum positioning error to be 46 km [45]. Hence, on the global scale, H5N1 cases can be considered points in space and time. A point pattern analysis of H5N1 cases is used to determine where and when cases are spread as well as which spatial and temporal scales are optimal for disease outbreak clustering. Choosing the right scale is critical in clustering analyses. Here we used exploratory spatial statistical techniques to examine the patterns of H5N1 outbreaks. We analyzed the data from (1) the spatial [13]; (2) the temporal [14]; and (3) the spatial-temporal perspectives [45].

**Figure 2:**
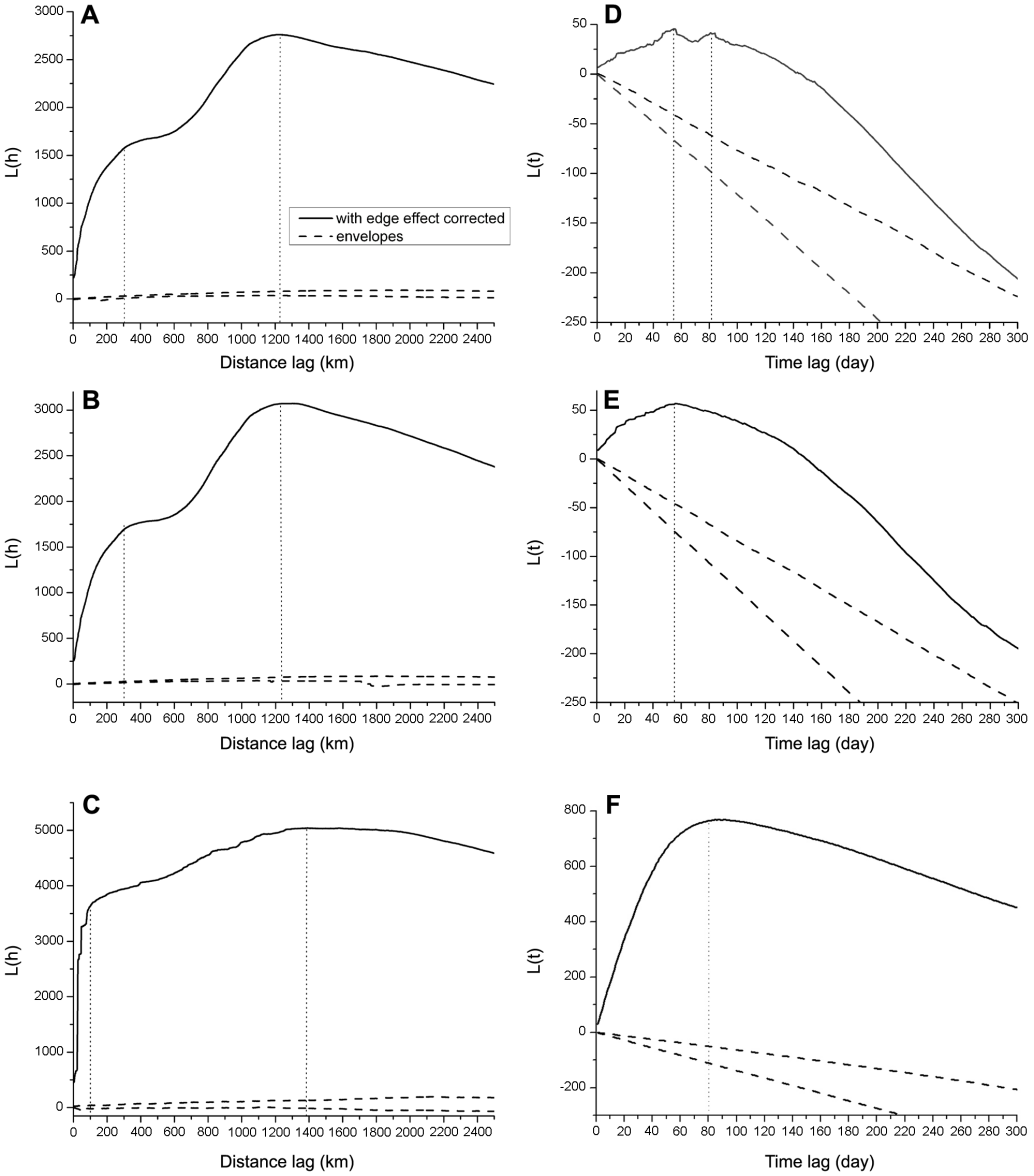
Spatial and temporal K function results. Spatial K function calculated for A) global infection; B) poultry; and C) wild birds. Temporal K function calculated for D) global infection; E) poultry, and F) wild birds.

**Figure 3:**
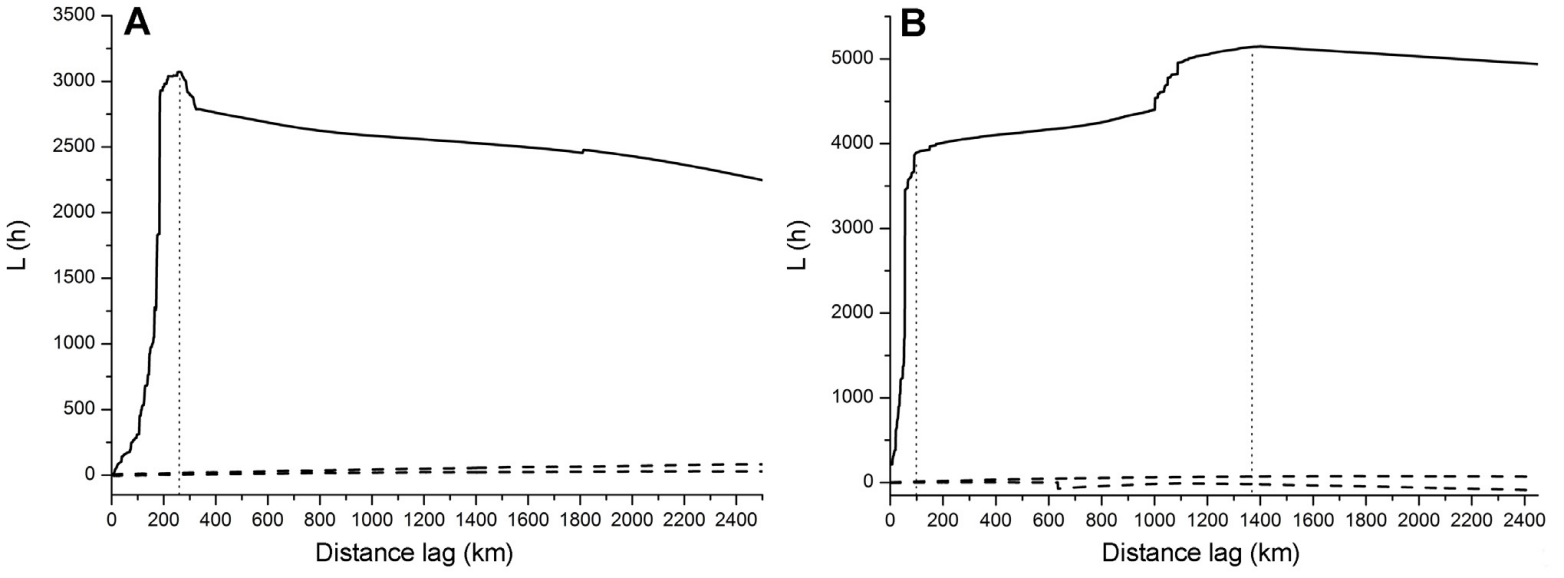
Spatial K function for A) city centers and B) important staging points of wild birds.

Ripley's K function is an effective approach to analyze point process data. Point process data contain locations and time of events. K function can be used to describe how the expected value of a point process changes over different spatial and/or temporal range of sizes (lag). K function value would be plotted against a series of increasing lags, and a peak value indicates an outbreak cluster emerging at the scale of the corresponding lag. Because the analysis is on a global scale, the spatial lag is in spherical distance (see the Methods for details of the calculation). This is different from the K function analysis based on a flat plane [45]. Avian flu primarily occurs on habitable land, so we excluded from our analysis inhabitable areas such as ocean and permanent ice cover derived from remotely sensed data [15]. Figure 2A shows the K function value calculated from the entire global dataset of H5N1 outbreaks. There are two peaks located at approximately 300 km and 1250 km. We calculated the K function of center points of residential areas, which are extracted from the 2003 Defense Meteorological Satellites Program (DMSP) nightlight data in “Version 2 DMSP-OLS Nighttime Lights Time Series” database [16] (see Method for a description of the processing procedure). The major peak is around 300 km. We also calculated the K function of important staging points of wild birds, which are crucial birds' habitats [17]. The peak spatial lag is around 1250–1300 km (Figure 3B). The spatial pattern of poultry is strikingly similar to that of the global patterns because a majority (88%) of the cases in the global dataset belongs to poultry cases (Figure 2B).

We suspect that the spatial lag for wild birds is related to migration distances of wild birds. Wild bird migration is considered as a mode of H5N1 transmission, referred to as the “bird migration” mode. The 300-km spatial lag corresponds to the average distance between major residential centers. Poultry are produced, transported, and consumed by people. Hence, the residential centers can be deemed as centers of poultry trade. This type of poultry trade represents a unique mode of H5N1 transmission by transportation, referred to as the “poultry transmission” mode.

In an epidemiological study, the intensity of disease outbreak cases varies with time. Thus, it is necessary to explore the temporal patterns in the global H5N1 cases using the extension of the K function. As can be seen in Figure 2d, the primary peak is located at 55 days, and a second peak is located at 80 days. The poultry cases exhibit a single peak at 55 days (Figure 2E), while the wild bird cases exhibit a single peak at 80 days (Figure 2F).

The 55-day peak suggests that H5N1 cases tend to concentrate during a period of 55 days. The temporal cycle may be explained by the passage of time from transportation, incubation, and the latent period up to the detection period. Under normal circumstances, the transportation time lasts from a few days to 2–3 weeks. The incubation period for the HPAI virus is highly variable, from 2 to 3 and up to 16 days. The persistence time of the virus also changes with the environment: it can survive in poultry houses for up to five weeks [18] and remain viable in feces for up to 32 days [19]. All these periods add up to about 30 to 60 days. An 80-day time lag may fit the migration cycle of migratory birds [20]. Migratory birds often take their spring or autumn migration for approximately three months. In general, only when birds settle at breeding or stopover sites, they could spread their self-carrying viruses when in contact with other birds or poultry.

**Figure 4:**
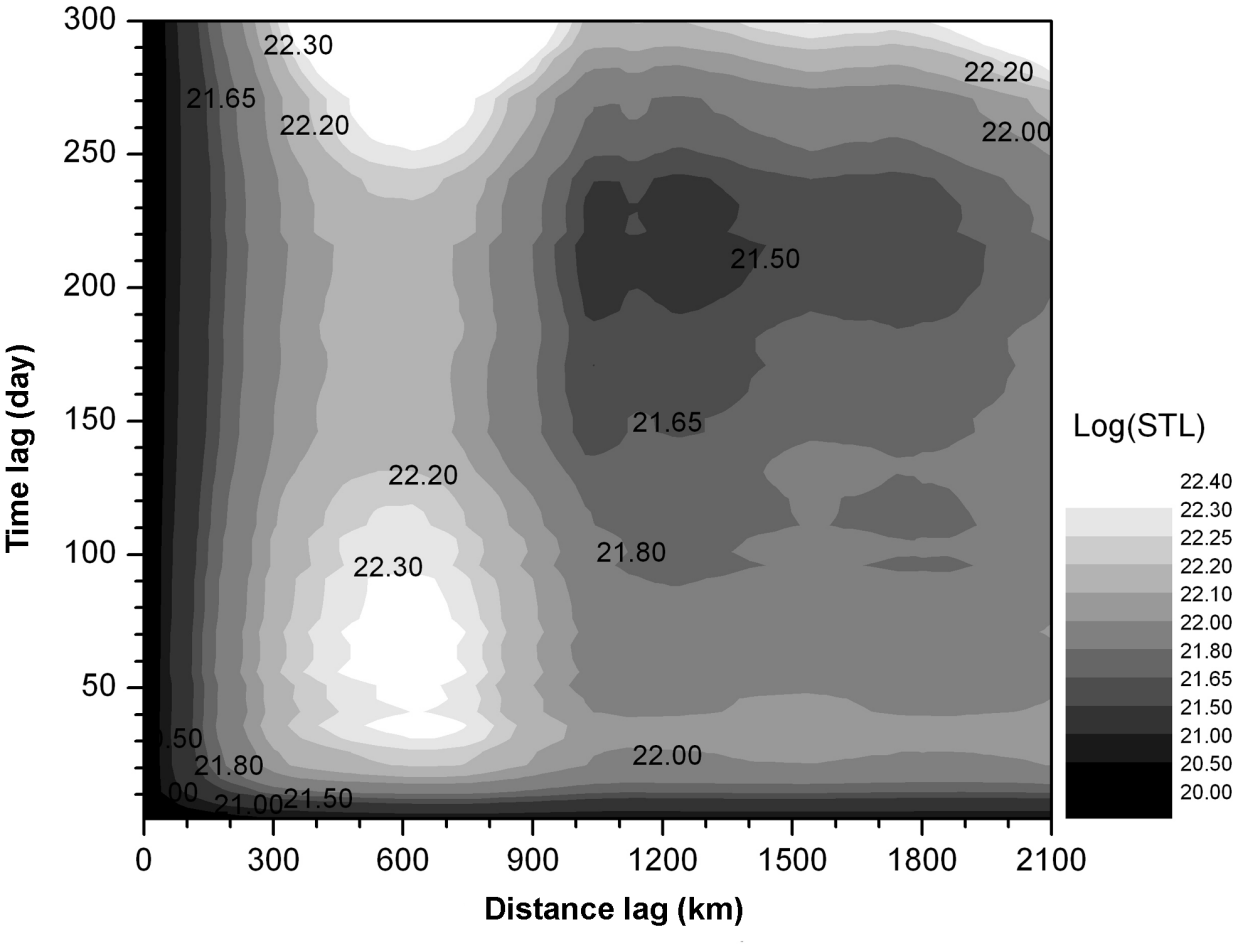
Contour map of Spatial-temporal K function.

The spatial-temporal pattern of H5N1 cases is illustrated by spatial-temporal K functions using a contour map (Figure 4). High values in the contour map indicate space-time clustering in the data. Only spatial-temporal patterns can realistically depict the spatial-temporal process of certain events of interest because spatial or temporal analysis alone may bring together irrelevant events that occur too far apart in space or in time to be captured by other means. Here, the most significant space-time peak involves a time period from 30 to 90 days and a spatial lag from 400 to 700 km. Interestingly, we find the lags to be similar to those of the separate spatial and temporal patterns of poultry, both close to the 300-km distance lag and covering the 55-day time lag. This implies that poultry abundance and density are responsible for the intensity of avian flu outbreaks. The two scales form the basis for choice of spatial and temporal scales in a subsequent spatial-temporal cluster analysis of the H5N1 outbreak cases. Poultry served as fuel and wild birds acted as fire seeds.

### Spatial-Temporal Cluster Analysis

From the spatial-temporal analysis of the H5N1 outbreaks, we found that they are clustered both in a short time and in a relatively small area (compared with the whole study area). Cluster patterns have an advantage: large quantity of points can be easily tracked in space and time. We used SaTScan statistics software to look for statistically significant spatial-temporal clusters based on a 400-km spatial scale and a 30-day time scale as mentioned above [21]. These two derived scales can help locate cluster centers and exclude those dependent cases around each cluster center. Phylogenetic analysis can then be applied to gene sequences at corresponding spatial-temporal clusters to identify the linkages between them. Possible spreading routes of H5N1 will then be derived.

### Phylogenetic Analysis

**Figure 5:**
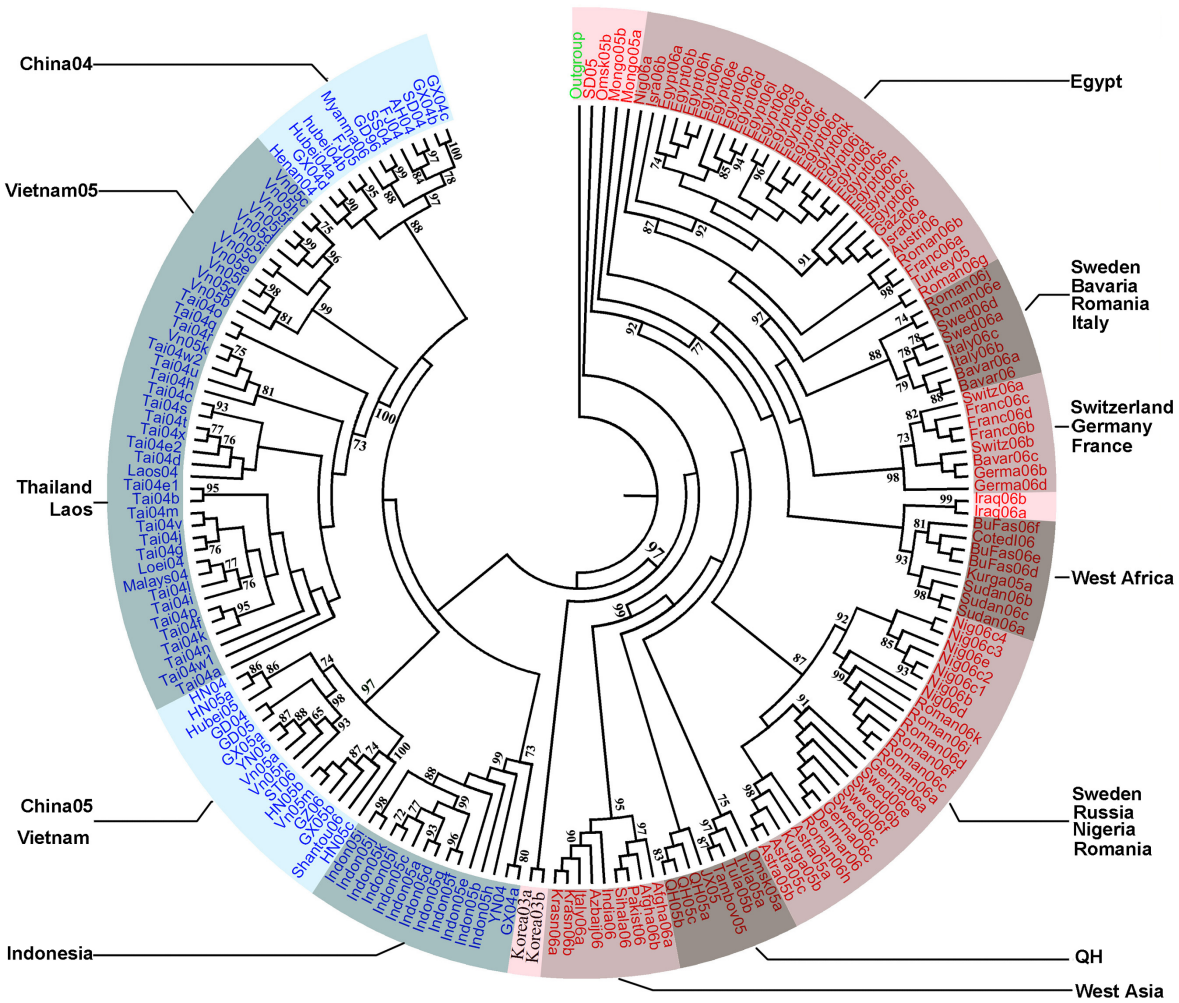
Phylogenetic organization based on HA gene sequences of H5N1. Phylogenetic relationships of 185 HA genes of representative influenza A virus isolated from 2003–2006 plus Gs/GD/96. Names of the genes were abbreviation of actual isolate names, which can be looked up in supplementary file “Table S1”. Letters stand for the isolated places, and numbers stand for the isolated year. Multiple sequence alignments were performed using Clustal W algorithm and Maximum Likelihood trees was reconstructed. Approximate Likelihood Ratio test values greater than 50% were marked at the branches.

From GenBank, we downloaded 185 influenza A H5N1 hemagglutinin (HA) gene sequences isolated during 2003 to 2006 with the same outbreak location and year as the clusters identified above. The A/Goose/guangdong/1/96 sequence was also downloaded for gene tree construction (Table S1, Figure 5). One caveat may be that, without detailed geographical and time information in those sequences, the clusters and gene sequences can not be matched exactly. To most sequences, the location and time information was only defined to country and year level. Strains located in large countries, like China, contain province-level information. We referenced the source articles of all sequences, and checked and implemented their detailed descriptions to the sequence database. With this accuracy improved dataset, it is relatively easier to match them with the geospatial data based on their spatial and temporal attributes. Mismatch during the georeferencing was unavoidable, but we attempted to minimize them to the minimum level. It is desirable that all available publicly sequences are precisely documented with exact location and time.

**Figure 6:**
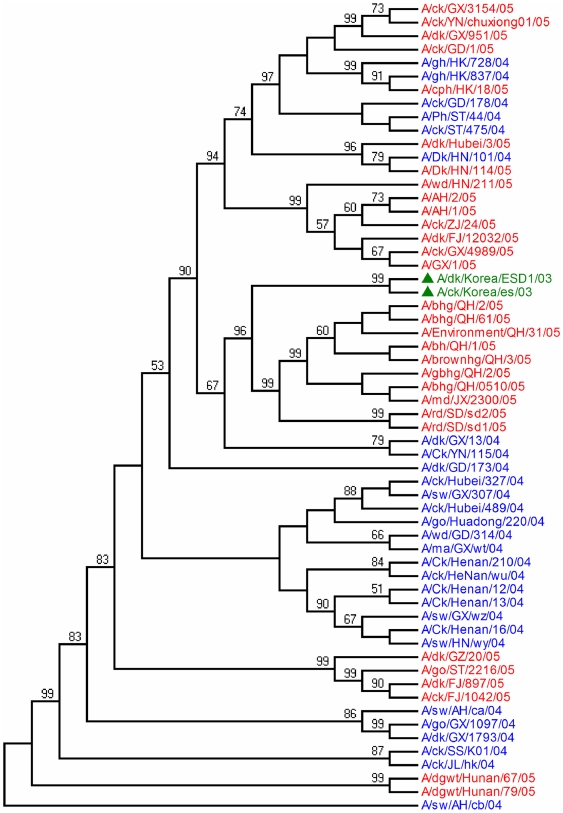
Phylogenetic relationships of H5N1 avian influenza strains in China. Phylogenetic tree based on HA genes of virus isolated in China were reconstructed as the method described in Figure 5.

From the perspective of geographic distribution, most sublineages in this phylogenetic tree comprise sequences from geographic adjacent areas, usually within one country. And they are labeled with the geographic region. Viruses isolated in China in 2004 are consistently grouped with A/Goose/Guangdong/1/96 (Figure 5). Although multiple reassortment events happened onward, and several replacements of predominant genotypes were observed from 1996 [48], [49], the Gs/GD/1/96 sublineage continued to be prevalent and circulating in South China in the past eight years. Whereas, viruses isolated in China in 2005 and 2006 formed an independent sublineage named China05, as a sister group to China04, suggesting a newly dominant group of viruses in China since 2005. This sublineage had been named by Smith et al. as Fujian-like [22]. Interestingly, the two isolates from South Korea in 2003 also fell in the China05 sublineage instead of the China04 (Figure 6). Thus, it is suspected that the viruses in the China05 sublineage may be reassorted or recombined from the Korea 2003 virus and the A/Goose/Guangdong/1/96 virus. Another noteworthy evidence is that all the Qinghai Lake isolates can be traced to one isolate from migratory ducks at Poyang Lake, north of JiangXi Province in February 2005. Qinghai Lake locates at northeastern Qinghai Province, and acts as one of the most important birds' breeding areas. All these suggest that migratory birds be responsible for the virus dissemination. Both Korea and South China are wintering regions in the East Asia flyway of migratory birds while East Siberia is the breeding region. When wintering birds carrying the Gs/GD/1/96 virus from China and those carrying the virus from Korea meet in East Siberia during the breeding season, their interaction could have caused the cross infection leading to a reassorted strain. From late 2004 to early 2005, migratory birds carried the reassorted viruses back to south China and were identified. The outbreak time and location of the virus at Poyang Lake and Qinghai Lake matched well with the pattern of bird migration. Thus, we speculate that the Qinghai Lake virus was a reassortant from the Fujian-like sublineage, possibly along with those from Southeast Asia as well [7].

In Southeast Asia, viruses in Vietnam have evolved over time into geographically distinct groups. Three isolates in North Vietnam in 2005 clustered into China04 sublineage. This suggests that the virus was likely introduced to Vietnam, most probably from Guangxi and Yunnan, China [2], [47]. Other isolates in South Vietnam, were clustered with those in Thailand and Laos in 2004. This introduction was likely from Thailand through Laos. All viruses from Indonesia in 2005 form a distinct sublineage suggesting their outbreaks are likely originated from a single introduction that spread throughout the country. Older viruses in the previous year from Yunnan and Guangxi of China, that are genetically clustered in the Indonesia group are suspected as the origin. Both Yunnan and Guangxi are located in southern china, while Guangxi shares its border with Vietnam, and Yunnan borders Myanmar, Laos, and Vietnam.

Besides the sublineages circulating in East and Southeast Asia, there is a dominant one containing isolates from Europe, Middle Asia and Africa (named as EMA sublineage). Many studies suggest that the origin of the EMA sublineage is Qinghai Lake [23]–[25]. The gene tree points to the evidence that the isolates in Southern Russia, such as Kurgan, Novosibirsk, Tula, and Astrakhan, are identical to the A/bar headed goose/Quinghai/1A/2005 strain. West Asia sublineage contains isolates from India, Afghanistan, Pakistan, Russia (Krasnodar), Azerbaijan and Italy. The close relationship between the Indian viruses and those in the Central Asia Flyway of migratory birds suggests that the viruses in West Asia may have been introduced through migratory birds from middle Siberia.

**Figure 7:**
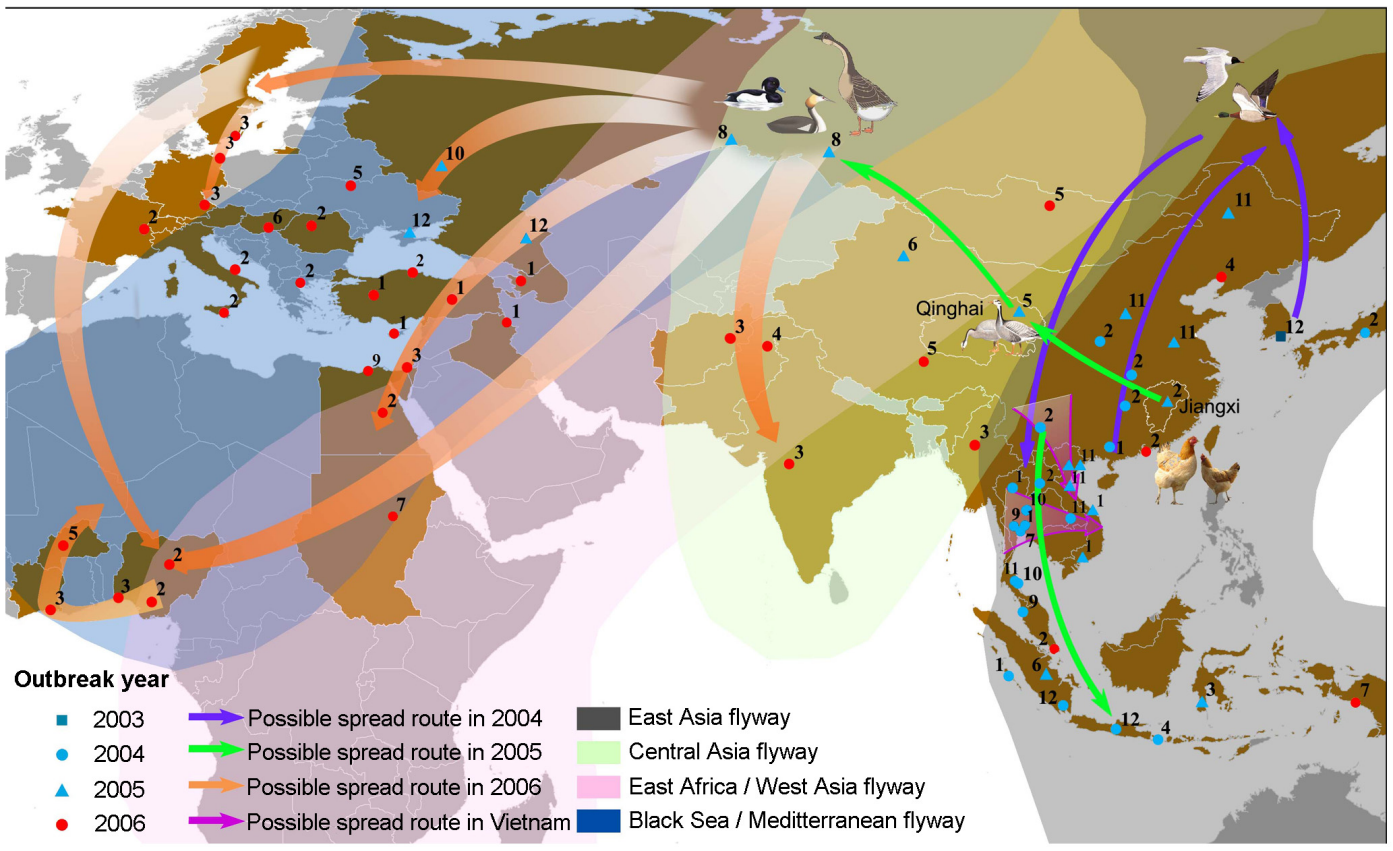
Representative spread routes of the H5N1 viruses in four years. Triangles and dots represent spatial-temporal cluster centers. The numbers on the triangles and dots are the reported months of the cases.

The sublineages of European countries reveal a western spread trend from Mongolia, Russia, Romania, to Northern European countries including Sweden and Denmark, and a southwest spread trend from those north European countries to West Africa. In all European sublineages, except Egypt and Romania, there is no solely contained stain from just one country. This means that the HPAI viruses in European countries have been highly mixed. They are related with each other but had reassorting characteristics. The Sweden, Germany, Italy sublineage contains strains from Romania, Italy, Sweden and Germany, revealing a longitudinal direction of virus spread. This spread route was supposed to be caused by a sudden westward movement of wild birds due to spills of Arctic cold in early 2006 [26]. In the West African sublineages, Sudan, Burkina Faso, Cote d'Ivoire are in a group with isolates from Kurgan and Russia. We speculate this sublineage was originated from Kurgan, then to central Asia (Iraq) and Europe, and finally reached Nigeria through the Black Sea-Meditterranean flyway. Isolations in Egypt are in the same group with those in Turkey and Romania with a 97% bootstrap rate implying that the viruses were introduced from west Russia to Turkey, then to Egypt. All the above suspected spread routes, along with the spatial-temporal cluster locations were drawn in Figure 7.

## Discussion

In this paper, we highlighted two transmission modes at the global scale through a spatial-temporal analysis: the poultry transmission mode and bird migration mode. From Figure 7, except for the introduction into North and South Vietnam which may be caused by poultry transmission, all remaining routes are most likely caused by the bird migration mode. This is because the spatial and temporal regularity can only be explained by routes of wild bird migration.

Spatially, the spread route coincides well with the migration flyways. Our study area contains four flyways among the eight main flyways in the world (Figure 7). The East Asia flyway connects the Far East of Russia, Eastern China and Southeast Asia. The Black Sea-Mediterranean Sea flyway mainly links Eastern Europe to wintering sites along the river systems crossing the Arabian Peninsula and the Nile. Birds from Western/Central Siberia and Central Asia fly along the East Africa-West Asia flyway to rest on the same wintering areas. The Central Asia flyway connects Siberia and Northern China to the southern part of North Asia, and Southwest Asia. The possible spread route in 2004, 2005 and 2006 matched well with these flyways. The cross continent and long distance transmission is not likely to be caused by poultry transportation and trading. Temporally, there is a strong correlation between the timing of the spreading of the H5N1 virus and the season of wild bird migration. As seen in Figure 7, outbreak always started in Southeast and East Asia during the spring migration period, and at the end of autumn migration, the virus activity in those regions intensifies. A clear spreading pattern can be observed outside Asia, where poultry management is more controlled and virus circulation history were not as frequent as they are in Southeast Asia. Cases occurred in Qinghai Lake in May and those in Siberia in August match the time of breeding season. After this period, birds began their autumn migration, which lasts from August to November. Different from the spring migration, in order to take care of the juveniles, birds fly slowly and stop more frequently, which favors wider spreading. Birds from Siberia migrate along the Black Sea-Meditterranean flyway. And during October and December, H5N1 viruses already had been spread to the warmer regions in the Black Sea area and the Mediterranean region. From December to the following March, western European and West Africa countries reported wild bird infection. Thus, we conclude that it is the wild birds that are the main agent for H5N1 virus dispersal.

Tremendous efforts have been put into the investigation on the source, the ways of introduction, and the evolution of H5N1 transmission [9]–[10], [27]. However, most analyses were conducted at regional scales and with a relatively short time span. Conclusions from such studies may lead to arbitrary and unilateral decision making. Gs/GD/1/96 sublineage has been circulating in China for almost ten years. After its reassortment into the Fujian-like virus, it was hypothesized to be facilitated by vaccination in China [22]. However, our analysis indicates that this mutation may be caused by cross-infection of wild birds in East Siberia.

We recommend that monitoring and surveillance are enhanced in three hinge sites in the way of H5N1 virus spread: East Siberia, Middle Siberia, as well as South China and the Southeast Asia region. The Russian Far East region is the most important breeding area in the East Asia flyway, with most H5N1 endemic countries falling into the wintering region of this flyway. However, less surveillance work is carried out in the Russian Far East because of its low population density. Thus, more efforts should be put to the virological surveillance, isolation and characterization of dead birds. Middle Siberia possesses the highest density and richest species of migratory birds. Three migration flyways cross-cover this region, but the migration patterns of wild birds are still not well understood there, making it hard to tract the complex migration movements and recognize the relationship among the infected wild birds in European, Africa and South Asia countries. Hence, more work is needed over these regions to study the spatial and temporal migration patterns of wild birds from a variety of avian orders. In order to eradicate the long circulating virus pool in South China and Southeast Asia, and reduce the possibility of cross infection between poultry and poultry, or wild birds and poultry, the governments of China and Southeast Asian countries should regulate their poultry market, especially the live poultry market and backyard poultry raising practices.

## Materials and Methods

### Spatial point pattern analysis

K function is originally defined as the following [28]:
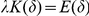
where 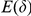 denotes the expectation of the number of events found within certain scale 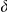, and 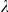 is the intensity of the events.

Therefore, a suitable estimate of spatial K function can be calculated by:
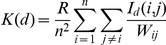
where 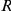 is the total area of the study, 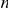 is the number of observed events, 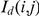 is an indicator function that takes value of 1 when the spherical distance between point 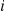, and point 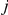 is less than 
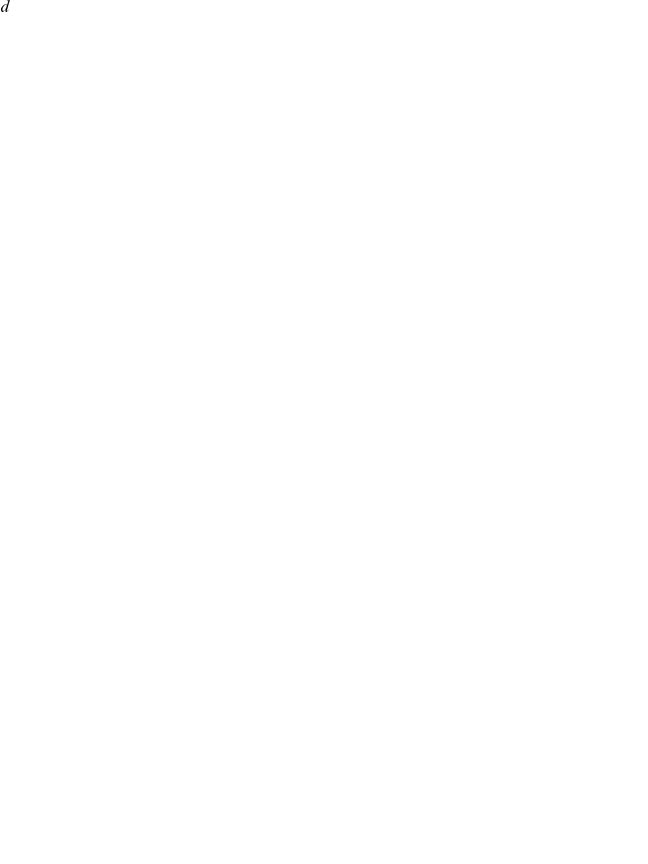
. 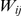 is the adjustment factor of the edge effect, which is the ratio between the area of a spherical circle of arc-radius d that excludes ocean and permanent ice cover and the area of the spherical circle. Because our analysis is done on a global scale, we work with spherical distance for any pair of points on the globe:
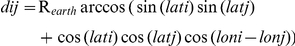



To assess the point pattern, one way is to compare 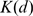 estimated from the observed data with 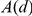 (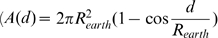), which is the area of the spherical circle of arc-radius 
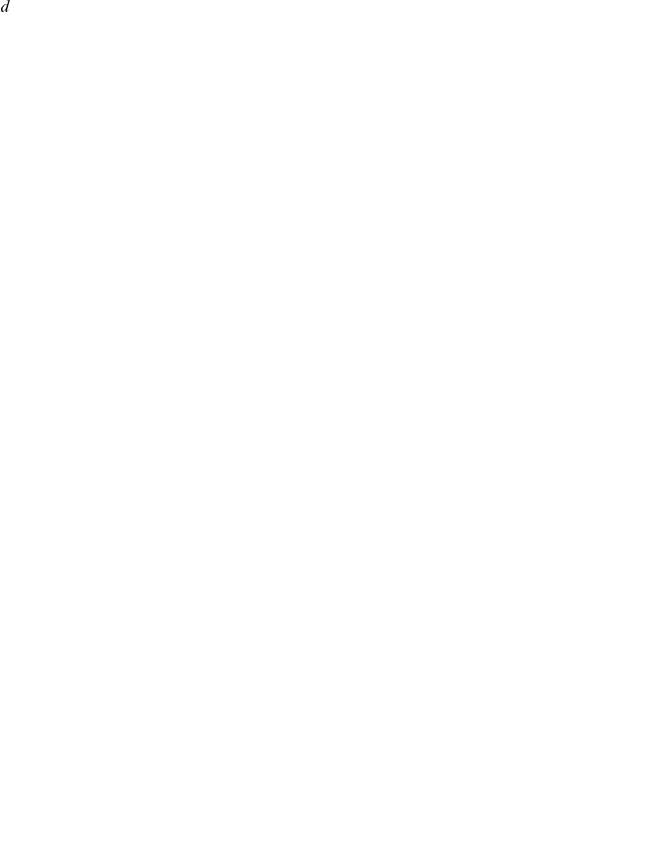
. In clustering, 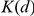 would be greater than 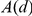, and less than 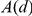 under regularity. We apply a transformation to 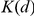 to have 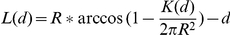, and to plot L(d) against d.

We calculated the distance from 1km and the bin size is 2km. In Figure 2 and 3, we only show curves before 2500km. Curves beyond 2500km show direct downward trend without much fluctuation.

The upper and lower bounds are determined by undertaking Monte Carlo simulations 99 times. For each simulation, we generate the same number of random points as the cases, and then calculate their K functions. To each lag, the upper and lower bounds are the minimum and maximum K values from 99 simulations.

### Temporal point pattern analysis

In the temporal K function, the time lag is 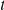, and intensity is calculated with respect to time. Consequently, the temporal K function takes the following form:
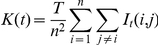
where, T is the time span from the earliest case to the latest case in our dataset. For convenience, we changed the Gregorian date to the Julian date. We apply 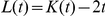 to the temporal K function for visualization purposes. To avoid the time edge effect in the analysis, we calculate from the first nth to the last nth point, which will only introduce the amplitude influence on the result (here we assign “n” as 300). Finally 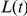 is plotted against 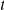. 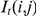 is an indicator function that takes value of 1 when the time interval between point 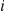 and point 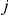 is less than 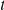.

### Spatial-temporal point pattern analysis

The spatial-temporal K function, 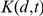 is defined as the expected number of events per unit of area and per unit of time. That is,
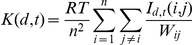
where, 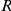, 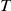, 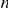 and 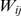 are as used above, and 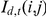 will be 1 if the distance and time intervals of point i and j are within 
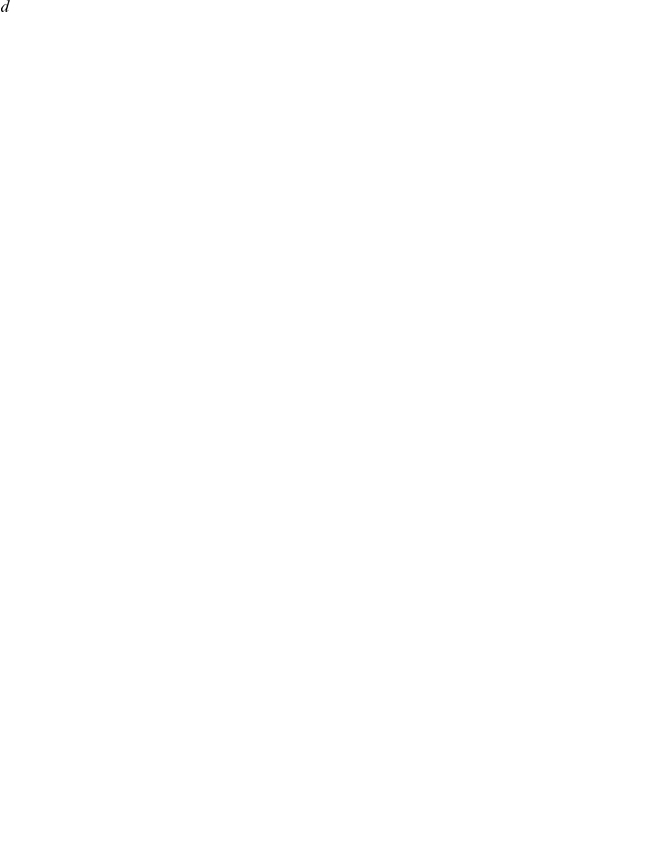
 and 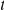, respectively.

If there is no spatial-temporal interaction, then 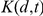 is simply the product of the spatial and temporal K-functions, 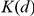 and 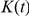, respectively. Thus, a test for space–time interaction may be based on this equation: 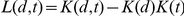.

### SaTScan and GIS used in exploring H5N1 spatial-temporal spread patterns

SaTScan is a freely available software program that can be used for cluster detection using the theory of scan statistics. To run SaTScan, two datasets are required. One is a coordinate file containing location identifiers (IDs) and x and y coordinates describing the location. The other one is a case file that contains location IDs, number of cases, and the date. The method uses a moving circular window with its center settled on any point of a map to scan the whole region. For each distinct window, a likelihood ratio is calculated. For circle 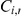, the likelihood ratio is
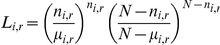
where 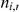 is the number of cases inside 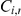, N is the total number of cases, and 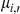 is the expected number of cases inside 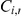, and i stands for the outbreak event.

The circle with the maximum likelihood is picked out and identified as the most likely cluster. To extend scan statistics to clustering in space and time, we use cylinders instead of circles, and the height of the cylinder represents time. The rest of the process is unchanged. The likelihood ratio is calculated for each cylinder and the scan statistic is the maximum likelihood ratio over all possible cylinders, again corresponding to the most likely cluster.

### DMSP night light data for extraction of major residential areas

The cleaned up average of the visible band data of the nighttime DMSP satellite images acquired in 2003 was used as a proxy measure of urban extent in this study. The dataset contains lights from cities, towns, and other sites with persistent lighting. Successful application in delineating the boundary of residential areas with nightlight data had been achieved [29]. Raw nightlight images have booming problem, which exaggerate and shift the extent of urban areas, even union two neighboring areas together. The images must be thresholded and different thresholds must be applied to different parts of the world with different level of economic development [30]. Overthresholding tends to eliminate areas with small extent. Hence, determining appropriate light thresholds for delineating residential areas remains a challenge. The simplest but efficient way to delineate the boundaries of residential areas is to visually compare the 1 kilometer resolution nightlight image with 30 meter resolution TM/ETM+ satellite images. Fourteen TM/ETM+ images acquired over different cities were chosen. Each image represents certain part of the world at different levels economic development. They were downloaded from the ESDI GLCF website [31]. The image acquisition date, mostly in 2000 and 2001, was close to the collection dates of the nightlight dataset. Detected urban areas with different thresholds from the DMSP image were compared with those urban areas delineated from TM/ETM+ images. Finally the most optimal threshold producing the nearest size and number of residential areas was chosen (Table 1). Boundaries of residential area overlaid on the corresponding TM/ETM+ images are shown in Figure S1. Our main purpose is to extract the center of each residential area, rather than their actual boundaries (Figure S2). Thus, a medium value, avoiding exaggeration or underestimation of the number of cities, is chosen for each continent.

**Table 1:** TM/TEM+ image acquisition date and DN threshold for each city.

Name			DN threshold		
Continent	City	Country	Continent	City	Image Date
Europe	Berlin	Germany	45	45	2000-08-14
	Bucoresti	Romania		45	2000-06-14
	Kobenhavn	Denmark		55	2000-05-08
	Ankara	Turkey		45–50	2000-05-10
	Bruxelles	Belgium		40	2001-07-03
Africa	Cairo	Egypt	25	60	2000-11-11
	Abujia	Nigeria		25	2001-12-27
	Yaounde	Cameroon		25	2000-05-18
Asia	Bangkok	Thailand	40	45	2000-11-02
	Hanoi	Vietnam		40	2000-11-04
	Beijing	China		55	2000-04-30
	Nanchang	China		35	2000-09-23
	Lanzhou	China		25	2001-07-14
Australia	Canberra	Australia	20	25	2001-04-25

### Phylogenetic analysis

Full length or partial length of hemagglutinin (HA) sequences were obtained from Genbank. Sequences were then aligned by CLUSTAL W algorithm [32] implemented in BioEdit v.7.0 [33]. Phylogenetic relationships of the HA were constructed by Neighbour-joining (NJ), Maximum Parsimony (MP) and Maximum Likelihood (ML) approaches (Figure S3, S4, 5). The best nucleotide substitution model was selected using the Akaike Information Criterion [34] and a hierarchical likelihood ratio [35] test in jModelTest v. 0.1.1 [36]. For our datasets, the General Time Reversible model [37] assuming a rate variation across sites according to a gamma-shaped distribution [38] with an estimated proportion of invariant sites [39] were selected by the above mentioned criteria. For NJ tree reconstructions, we used composite maximum likelihood algorithms implemented in MEGA v. 4.0 [40] to estimate the transversion/ transition bias and nucleotide substitution patterns. We assumed that substitution rate varies among sites and substitution pattern heterogeneous among lineages. Similarly, alignments were examined and investigated by an MP approach with heuristic search in MEGA. Tree reliabilities were tested with 1000 bootstrap replicates to yield a majority consensus tree. For ML tree reconstruction, we used a web server (http://www.atgc-montpellier.fr/phyml/) to perform PhyML [41]. The heuristic searching strategy for the best topology was started via five random BIONJ trees and those trees were moved by nearest-neighbour interchange (NNI) and subtree pruning and regrafting (SPR) approaches. Clade support for the phylogenetic inferences was estimated by the approximate Likelihood Ratio Test [42] using a Shimodaira-Hasegawa-like procedure in PhyML. Numerous simulations showed that aLRT estimation for the probability of a branch is better than bootstrapping [43]. For all tree-based analysis, bootstrapping and aLRT values of the nodes in the consensus tree were estimated. The final consensus trees were visualized and edited in TreeView [43] and FigTree v.1.2.3 [44]. The aLRT and bootstrap value ≥50% was regarded as high support. Sublineages with high support in ML, NJ and MP trees are discussed in this paper. Since the phylogenetic organizations of the three trees are identical, we only used the ML tree for illustration. NJ and MP trees can be found in supplementary files.

## Supporting Information

Figure S1 Fourteen TM/TEM+ images of selected cities with derived settlement boundaries on them.
(8.44 MB TIF)

Figure S2 Settlement boundaries derived from DMSP night lights using thresholding method.
(2.36 MB TIF)

Figure S3 Phylogenetic organization based on HA gene sequences of H5N1 using Neighbour-joining method.
(2.11 MB TIF)

Figure S4 Phylogenetic organization based on HA gene sequences of H5N1 using Maximum Parsimony method.
(1.83 MB TIF)

Table S1 Information of H5N1 hemagglutinin (HA) sequences collected from Genbank.
(0.33 MB DOC)
