# Supplementary figures and images for: Combining Spatial-Temporal and Phylogenetic Analysis Approaches for Improved Understanding on Global H5N1 Transmission
Source: PLoS One. 2010 Oct 22;5(10):e13575. doi: 10.1371/journal.pone.0013575 (PMC2962646; doi:10.1371/journal.pone.0013575)

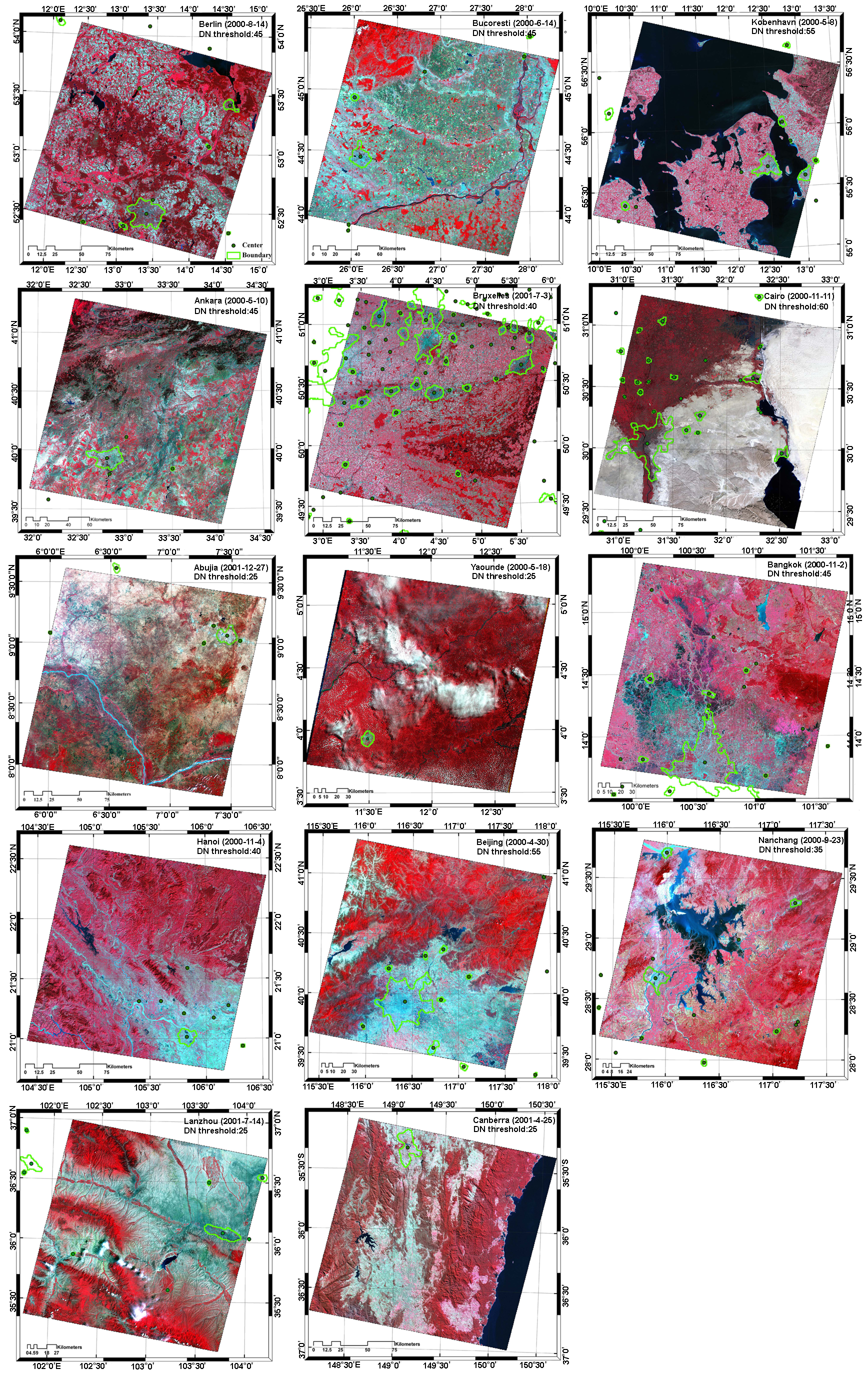

Supplement: Figure S1 — Fourteen TM/TEM+ images of selected cities with derived settlement boundaries on them. (8.44 MB TIF) [file pone.0013575.s001.tif]

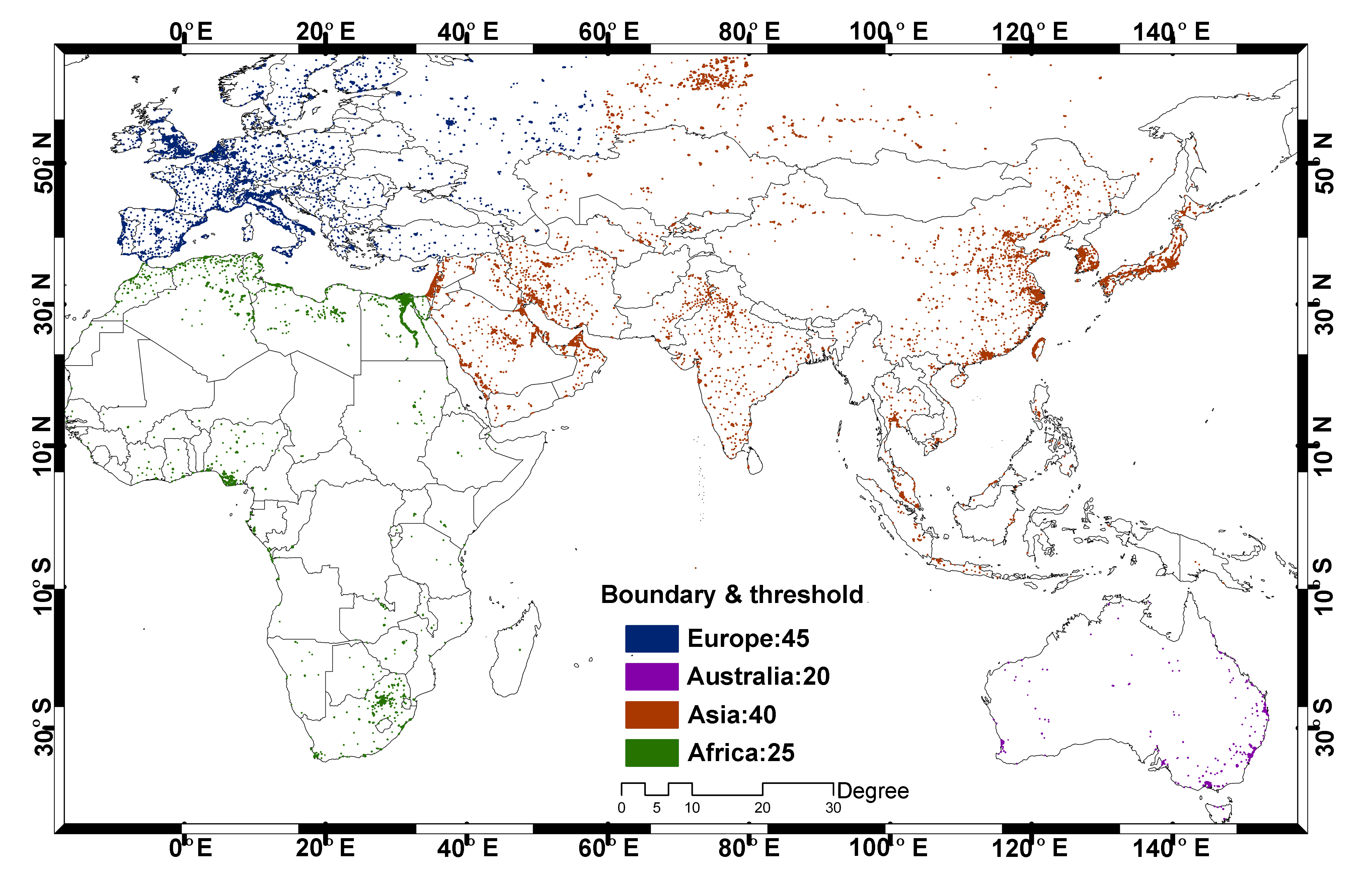

Supplement: Figure S2 — Settlement boundaries derived from DMSP night lights using thresholding method. (2.36 MB TIF) [file pone.0013575.s002.tif]

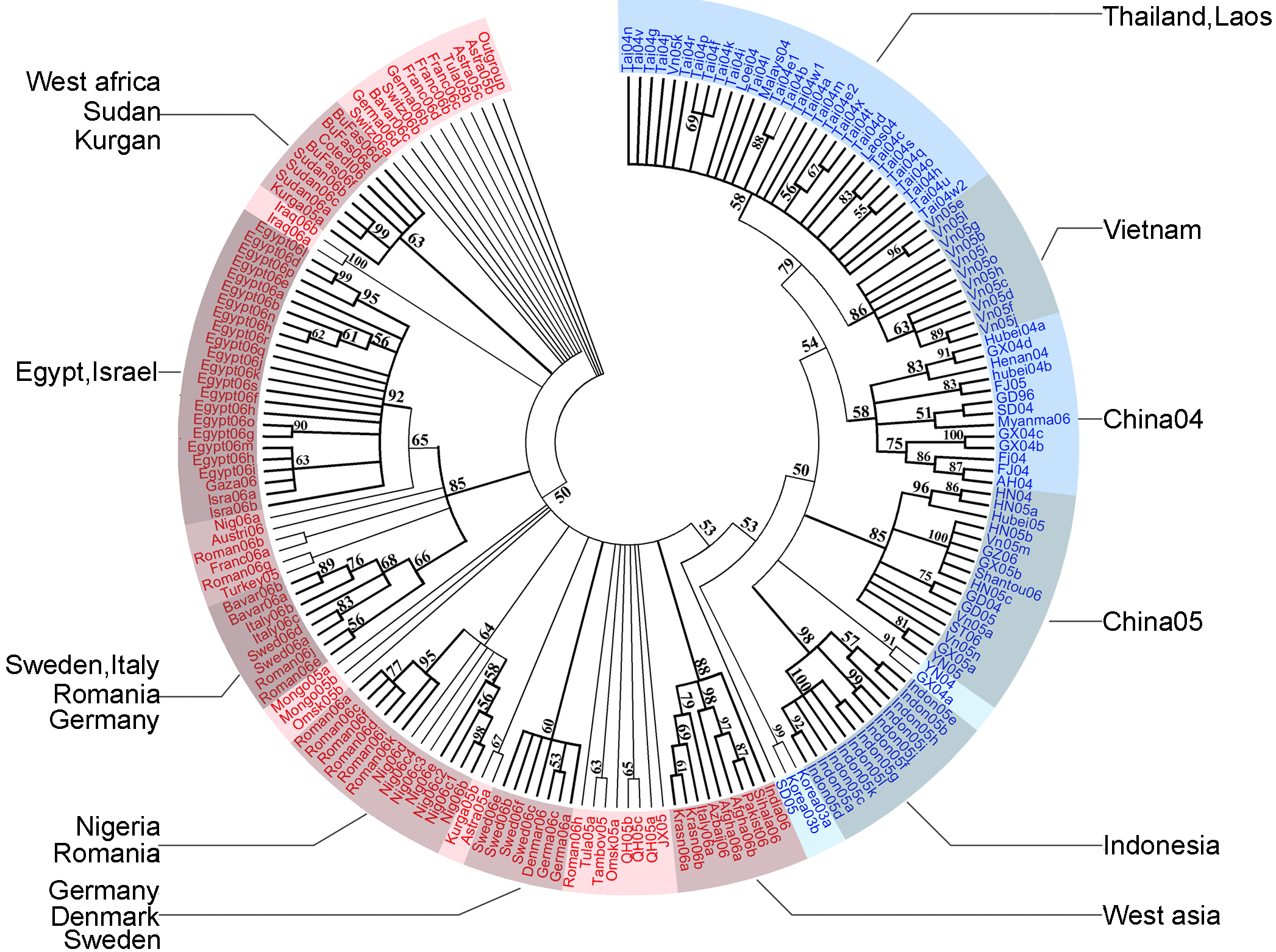

Supplement: Figure S3 — Phylogenetic organization based on HA gene sequences of H5N1 using Neighbour-joining method. (2.11 MB TIF) [file pone.0013575.s003.tif]

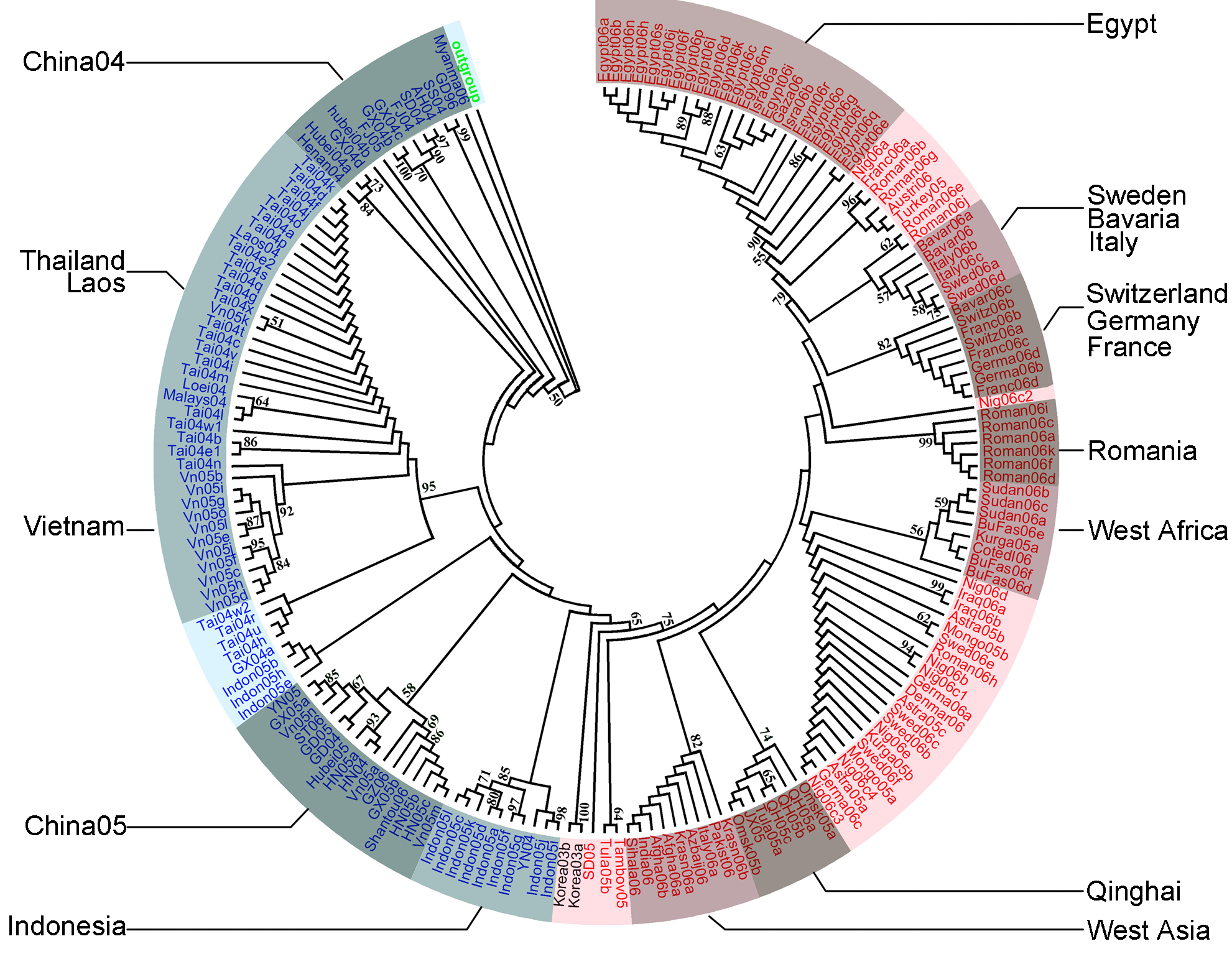

Supplement: Figure S4 — Phylogenetic organization based on HA gene sequences of H5N1 using Maximum Parsimony method. (1.83 MB TIF) [file pone.0013575.s004.tif]
