# Supplementary material for: Combining Spatial-Temporal and Phylogenetic Analysis Approaches for Improved Understanding on Global H5N1 Transmission
Source: PLoS One. 2010 Oct 22;5(10):e13575. doi: 10.1371/journal.pone.0013575 (PMC2962646; doi:10.1371/journal.pone.0013575)
Supplement: Table S1 — Information of H5N1 hemagglutinin (HA) sequences collected from Genbank. (0.33 MB DOC) [file pone.0013575.s005.doc]

| **Genbank accession number** | **Isolate name** | **abbreviation** | **Isolated country** | **Isolated place** | **Isolated date** |
| --- | --- | --- | --- | --- | --- |
| CY016787 | A/chicken/Afghanistan/1207/2006(H5N1) | Afgha06a | Afghanistan |  | 2006 |
| CY020637 | A/chicken/Afghanistan/1573-92/2006(H5N1) | Afgha06b | Afghanistan |  | 2006 |
| EF395845 | A/swan/Austria/216/2006(H5N1) | Austri06 | Austria |  | 2006 |
| EU146871 | A/Azerbaijan/006-207/2006(H5N1) | Azbaij06 | Azerbaijan |  | 2006 |
| AM400971 | A/Hooded vulture/Burkina Faso/1/2006(H5N1) | BuFas06f | Burkina Faso | Ouagadougou | 2006-4-3 |
| EF090647 | A/guinea fowl/Burkina Faso/5346-26/2006(H5N1) | BuFas06e | Burkina Faso |  | 2006 |
| EU277833 | A/chicken/Burkina Faso/1347-16/2006(H5N1) | BuFas06d | Burkina Faso |  | 2006 |
| AF144305.1 | A/Goose/Guangdong/1/96 | GD96 | China | Guangdong | 1996 |
| AY651365 | A/Dk/HN/101/2004(H5N1) | HN04 | China |  | 2004-1-1 |
| AY651372 | A/Ck/YN/115/2004(H5N1) | YN04 | China |  | 2004 |
| AY684706 | A/chicken/Hubei/327/2004(H5N1) | Hubei04a | China |  | 2004 |
| AY737296 | A/chicken/Guangdong/178/04(H5N1) | GD04 | China |  | 2004 |
| AY830774 | A/chick/Macheng/2004(H5N1) | Hubei04b | China |  | 2004 |
| AY950231 | A/Chicken/Henan/210/2004 (H5N1) | Henan04 | China |  | 2004 |
| AY950236 | A/swan/Guangxi/307/2004 (H5N1) | GX04d | China |  | 2004 |
| DQ095618 | A/Bar-headed Goose/Qinghai/61/05(H5N1) | QH05c | China |  | 2005 |
| DQ095630 | A/Duck/Hunan/114/05(H5N1) | HN05a | China |  | 2005 |
| DQ100556 | A/black-headed gull/Qinghai/1/2005(H5N1) | QH05b | China |  | 2005 |
| DQ137873 | A/bar-headed goose/Qinghai/0510/05(H5N1) | QH05a | China |  | 2005 |
| DQ188906 | A/black bulbul/Fujian/439/04(H5N1) | FJ04 | China |  | 2004 |
| DQ188908 | A/slaty-backed gull/Shandong/59/04(H5N1) | SD04 | China |  | 2004 |
| DQ320876 | A/chicken/Fujian/1042/2005(H5N1) | FJ05 | China |  | 2005 |
| DQ320881 | A/goose/Guangxi/1097/2004(H5N1) | GX04c | China |  | 2004 |
| DQ320887 | A/duck/Guangxi/1793/2004(H5N1) | GX04b | China |  | 2004 |
| DQ320921 | A/migratory duck/Jiangxi/2300/2005(H5N1) | JX05 | China |  | 2005 |
| DQ366338 | A/duck/Guangxi/13/2004(H5N1) | GX04a | China |  | 2004 |
| DQ520856 | A/duck/Hubei/3/2005(H5N1) | Hubei05 | China |  | 2005 |
| DQ767725 | A/chicken/Shandong/K01/2004(H5N1) | SD04 | China |  | 2004 |
| DQ842487 | A/goose/Guangzhou/471/2006(H5N1) | GZ06 | China |  | 2006 |
| DQ992718 | A/chicken/Guangxi/3154/2005(H5N1) | GX05a | China |  | 2005 |
| DQ992734 | A/chicken/Guangxi/4989/2005(H5N1) | GX05b | China |  | 2005 |
| DQ992782 | A/pheasant/Shantou/2239/2006(H5N1) | ST06 | China |  | 2006 |
| DQ992785 | A/chicken/Shantou/3840/2006(H5N1) | Shantou06 | China |  | 2006 |
| DQ992932 | A/duck/Hunan/5191/2005(H5N1) | HN05b | China |  | 2005 |
| DQ997392 | A/swine/Anhui/ca/2004(H5N1) | AH04 | China |  | 2004 |
| EU329186 | A/wild duck/Hunan/211/2005(H5N1) | HN05c | China |  | 2005 |
| EU420038 | A/raccoon dog/Shandong/sd1/2005(H5N1) | SD05 | China |  | 2005 |
| EU635874 | A/chicken/Yunnan/chuxiong01/2005(H5N1) | YN05 | China |  | 2005 |
| EU874899 | A/chicken/Guangdong/1/2005(H5N1) | GD05 | China |  | 2005 |
| CY016811 | A/chicken/Cote d'Ivoire/1787-34/2006(H5N1) | CotedI06 | Cote d'Ivoire |  | 2006 |
| EF523687 | A/buzzard/Denmark/6370/06(H5N1) | Denmar06 | Denmark |  | 2006 |
| CY020653 | A/turkey/Egypt/2253-2/2006(H5N1) | Egypt06h | Egypt | menia | 2006-2-13 |
| DQ447199 | A/chicken/Egypt/960N3-004/2006(H5N1) | Egypt06k | Egypt | giza | 2006-2-13 |
| DQ862001 | A/duck/Egypt/2253-1/2006(H5N1) | Egypt06t | Egypt |  | 2006-2-12 |
| DQ862002 | A/duck/Egypt/2253-3/2006(H5N1) | Egypt06c | Egypt | giza | 2006-5-2 |
| EF042622 | A/chicken/Egypt/10845-NAMRU3/2006(H5N1) | Egypt06s | Egypt |  | 2006 |
| EF441276 | A/chicken/Egypt/1078-NAMRU3/2006(H5N1) | Egypt06p | Egypt |  | 2006-12-1 |
| EF441278 | A/chicken/Egypt/1080-NAMRU3/2006(H5N1) | Egypt06q | Egypt | denofia | 2006-9-28 |
| EF441279 | A/chicken/Egypt/1081-NAMRU3/2006(H5N1) | Egypt06r | Egypt | damietta | 2006-11-22 |
| EF469658 | A/goose/Egypt/13009N3-SM2/2006(H5N1) | Egypt06g | Egypt | suphanburi | 2006 |
| EU146866 | A/chicken/Egypt/3/2006(H5N1) | Egypt06j | Egypt |  | 2006 |
| EU183325 | A/duck/Egypt/F5/2006(H5N1) | Egypt06d | Egypt |  | 2006 |
| EU183327 | A/chicken/Egypt/R1/2006(H5N1) | Egypt06l | Egypt |  | 2006 |
| EU372943 | A/chicken/Egypt/06207-NLQP/2006(H5N1) | Egypt06a | Egypt |  | 2006-2-19 |
| EU372944 | A/chicken/Egypt/06459-3-NLQP/2006(H5N1) | Egypt06i | Egypt | sharkia | 2006-3-13 |
| EU372945 | A/chicken/Egypt/06495-3-NLQP/2006(H5N1) | Egypt06m | Egypt | denofia | 2006-3-17 |
| EU372946 | A/chicken/Egypt/06541-NLQP/2006(H5N1) | Egypt06n | Egypt | sharkia | 2006-3-20 |
| EU372947 | A/chicken/Egypt/06959-NLQP/2006(H5N1) | Egypt06o | Egypt | cairo | 2006-11-27 |
| EU599298 | A/domestic poultry/Egypt/906089/2006(H5N1) | Egypt06e | Egypt |  | 2006 |
| EU719116 | A/egret/Egypt/1162-NAMRU3/2006(H5N1) | Egypt06f | Egypt |  | 2006 |
| FJ472343 | A/chicken/Qalubia/1/2006(H5N1) | Egypt06b | Egypt |  | 2006 |
| AM498628 | A/common pochard/France/06167-2.1/2006(H5N1) | Franc06a | France | La Dombes | 2006-2-16 |
| AM498629 | A/turkey/France/06222-1.1/2006(H5N1) | Franc06d | France | La Dombes | 2006-2-23 |
| AM498631 | A/greylag goose/France/06310/2006(H5N1) | Franc06b | France | La Dombes | 2006-3-7 |
| AM498632 | A/mute swan/France/06631a/2006(H5N1) | Franc06c | France | La Dombes | 2006-3-11 |
| EF532632 | A/duck/Gaza/760/2006(H5N1) | Gaza06 | Gaza |  | 2006 |
| AM231714 | A/swan/Germany/R65/2006(H5N1) | Germa06c | Germany |  | 2006 |
| AM403473 | A/eagle owl/Germany/R1166/06(H5N1) | Germa06b | Germany | south | 2006 |
| AM408209 | A/cormorant/Germany/R292/06(H5N1) | Germa06a | Germany | north | 2006 |
| AM408216 | A/tufted duck/Germany/R1240/06(H5N1) | Germa06d | Germany | central | 2006 |
| DQ659679 | A/common bussard/Bavaria/2/2006(H5N1) | Bavar06b | Germany |  | 2006 |
| EF165049 | A/buzzard/Bavaria/5/2006(H5N1) | Bavar06c | Germany |  | 2006 |
| EF165056 | A/mute swan/Bavaria/12/2006(H5N1) | Bavar06a | Germany |  | 2006 |
| EF362426 | A/chicken/India/NIV33491/06(H5N1) | India06 | India06 |  | 2006 |
| DQ497642 | A/chicken/Malang/BBVet-IV/2004(H5N1) | Indon05h | Indonesia |  | 2005 |
| DQ497657 | A/chicken/Jembrana/BPPV6/2004(H5N1) | Indon05e | Indonesia |  | 2005 |
| DQ497669 | A/chicken/Tarutung/BPPVI/2005(H5N1) | Indon05l | Indonesia |  | 2005 |
| EU124083 | A/Chicken/Taput/BBPV1-576/2005(H5N1) | Indon05k | Indonesia |  | 2005 |
| EU124090 | A/Turkey/Langkat/BBPVI/2005(H5N1) | Indon05f | Indonesia |  | 2005 |
| EU124091 | A/Chicken/Deli Derdang/BBPVI/2005(H5N1) | Indon05c | Indonesia |  | 2005 |
| EU124095 | A/Chicken/Agam/BBPVI/2005(H5N1) | Indon05a | Indonesia |  | 2005 |
| EU124097 | A/Chicken/Murao Jambi/BBPV-II/2005(H5N1) | Indon05i | Indonesia |  | 2005 |
| EU124098 | A/Chicken/Duma/BBPV-II/2005(H5N1) | Indon05d | Indonesia |  | 2005 |
| EU124103 | A/Chicken/Sembawa/BPPV-III/2005(H5N1) | Indon05j | Indonesia |  | 2005 |
| EU124105 | A/Duck/Madiun/BBVW1358/2005(H5N1) | Indon05g | Indonesia |  | 2005 |
| EU124107 | A/Duck/Bufeleng/BPPV1/2005(H5N1) | Indon05b | Indonesia |  | 2005 |
| DQ435202 | A/human/Iraq/207-NAMRU3/2006(H5N1) | Iraq06a | Iraq |  | 2006 |
| EU146876 | A/Iraq/659/2006(H5N1) | Iraq06b | Iraq |  | 2006 |
| EF532625 | A/turkey/Israel/365/2006(H5N1) | Isra06b | Israel |  | 2006 |
| EF532629 | A/chicken/Israel/625/2006(H5N1) | Isra06a | Israel |  | 2006 |
| CY020349 | A/cygnus olor/Italy/808/2006(H5N1) | Italy06b | Italy |  | 2006 |
| DQ412997 | A/Cygnus olor/Italy/742/2006(H5N1) | Italy06a | Italy |  | 2006 |
| DQ449031 | A/mallard/Italy/835/2006(H5N1) | Italy06c | Italy |  | 2006 |
| AY676035 | A/chicken/Korea/ES/03(H5N1) | Korea03a | korea |  | 2003 |
| AY676036 | A/duck/Korea/ESD1/03(H5N1) | Korea03b | korea |  | 2003 |
| EF541415 | A/chicken/Laos/44/2004(H5N1) | Laos04 | Laos | kanchanaburi | 2004 |
| DQ320935 | A/quail/Malaysia/6309/2004(H5N1) | Malays04 | Malaysia |  | 2004 |
| AB233319 | A/bar-headed goose/Mongolia/1/05(H5N1) | Mongo05b | Mongolia |  | 2005 |
| AB233321 | A/whooper swan/Mongolia/4/05(H5N1) | Mongo05a | Mongolia |  | 2005 |
| AB474081 | A/chicken/Pyigyitagon/204/2006(H5N1) | Myanma06 | Myanmar |  | 2006 |
| AM503005 | A/chicken/Nigeria/OD8/2006(H5N1) | Nig06b | Nigeria |  | 2006 |
| EF631175 | A/chicken/Nigeria/VRD457/2006(H5N1) | Nig06c2 | Nigeria |  | 2006 |
| EF631180 | A/chicken/Nigeria/VRD403/2006(H5N1) | Nig06c1 | Nigeria |  | 2006 |
| EF631183 | A/guinea fowl/JWP/Nigeria/VRD252/2006(H5N1) | Nig06d | Nigeria |  | 2006 |
| EF631184 | A/pigeon/Nigeria/VRD370/2006(H5N1) | Nig06e | Nigeria |  | 2006 |
| EF631186 | A/turkey/Nigeria/VRD345/2006(H5N1) | Nig06c3 | Nigeria |  | 2006 |
| EF631187 | A/vulture/Nigeria/VRD184/2006(H5N1) | Nig06c4 | Nigeria |  | 2006 |
| EU697217 | A/chicken/Nigeria/228-5/2006(H5N1) | Nig06a | Nigeria |  | 2006 |
| CY037770 | A/chicken/Sihala/NARC3303.4/2006(H5N1) | Sihala06 | Pakistan |  | 2006 |
| EU401795 | A/goose/Lahore-Pakistan/NARC-3321/4/2006(H5N1) | Pakist06 | Pakistan | gharbiva | 2006 |
| FJ785124 | A/chicken/Dolj/RO-AI-103/2006(H5N1) | Roman06h | Romania |  | 2006 |
| FJ785125 | A/wild goose/Braila/RO-AI-074/2006(H5N1) | Roman06b | Romania |  | 2006 |
| FJ785126 | A/chicken/Dambovita/RO-AI-131/2006(H5N1) | Roman06g | Romania |  | 2006 |
| FJ785127 | A/Guinea fowl/Constanta/RO-AI-159/2006(H5N1) | Roman06e | Romania |  | 2006 |
| FJ785130 | A/wild goose/Olt/RO-AI-288/2006(H5N1) | Roman06j | Romania |  | 2006 |
| FJ785131 | A/chicken/Brasov/RO-AI-350/2006(H5N1) | Roman06c | Romania |  | 2006 |
| FJ785135 | A/chicken/Covasna/RO-AI-379/2006(H5N1) | Roman06f | Romania |  | 2006 |
| FJ785136 | A/chicken/Valcea/RO-AI-479/2006(H5N1) | Roman06k | Romania |  | 2006 |
| FJ785137 | A/domestic duck/Bucharest/RO-AI-482/2006(H5N1) | Roman06d | Romania |  | 2006 |
| FJ785140 | A/chicken/Bacau/RO-AI-527/2006(H5N1) | Roman06a | Romania |  | 2006 |
| FJ785141 | A/chicken/Giurgiu/RO-AI-575/2006(H5N1) | Roman06i | Romania |  | 2006 |
| DQ320137 | A/swan/Astrakhan/1/2005(H5N1) | Astra05b | Russia |  | 2005 |
| DQ323672 | A/chicken/Kurgan/3/2005(H5N1) | Kurga05a | Russia |  | 2005 |
| DQ363923 | A/Cygnus olor/Astrakhan/Ast05-2-7/2005(H5N1) | Astra05a | Russia |  | 2005 |
| DQ449640 | A/duck/Kurgan/08/2005(H5N1) | Kurga05b | Russia |  | 2005 |
| DQ840519 | A/chicken/Tula/Russia/Oct-5/2005(H5N1) | Tula05b | Russia |  | 2005 |
| DQ840533 | A/swan/Astrakhan/Russia/Nov-2/2005(H5N1) | Astra05c | Russia |  | 2005 |
| DQ864716 | A/chicken/Tambov/570-2/05(H5N1) | Tambov05 | Russia |  | 2005 |
| DQ864718 | A/chicken/Krasnodar/199/06(H5N1) | Krasn06b | Russia |  | 2006 |
| DQ864721 | A/wild duck/Omsk/103-01/05(H5N1) | Omsk05b | Russia |  | 2005 |
| EF205155 | A/chicken/Omsk/14/05(H5N1) | Omsk05a | Russia |  | 2005 |
| EF205159 | A/chicken/Krasnodar/123/06(H5N1) | Krasn06a | Russia |  | 2006 |
| EF205160 | A/chicken/Tula/4/05(H5N1) | Tula05a | Russia |  | 2005 |
| CY016300 | A/chicken/Sudan/1784-10/2006(H5N1) | Sudan06a | sudan |  | 2006 |
| CY021389 | A/chicken/Sudan/2115-10/2006(H5N1) | Sudan06c | sudan |  | 2006 |
| DQ862003 | A/chicken/Sudan/1784/2006(H5N1) | Sudan06b | sudan |  | 2006 |
| EU889071 | A/eagle owl/Sweden/V1218/2006(H5N1) | Swed06c | Sweden |  | 2006 |
| EU889073 | A/tufted duck/Sweden/V998/2006(H5N1) | Swed06e | Sweden |  | 2006 |
| EU889076 | A/canada goose/Sweden/V978/2006(H5N1) | Swed06f | Sweden |  | 2006 |
| EU889077 | A/mute swan/Sweden/V827/2006(H5N1) | Swed06b | Sweden |  | 2006 |
| EU889078 | A/herring gull/Sweden/V1116/2006(H5N1) | Swed06a | Sweden |  | 2006 |
| EU889081 | A/tufted duck/Sweden/V599/2006(H5N1) | Swed06d | Sweden |  | 2006 |
| EF110518 | A/goosander/Switzerland/V82/06 (H5N1) | Switz06a | Switzerland |  | 2006 |
| AB440324 | A/chicken/Suphanburi/1/2004(H5N1) | Tai04w1 | Thailand |  | 2004-1-21 |
| AB440325 | A/duck/Angthong/72/2004(H5N1) | Tai04e1 | Thailand |  | 2004-1-23 |
| AB450550 | A/chicken/Kalasin/NIAH316/2004(H5N1) | Tai04h | Thailand |  | 2004-1-30 |
| AB450552 | A/chicken/Kohn Kaen/NIAH330/2004(H5N1) | Tai04j | Thailand |  | 2004-1-30 |
| AB450553 | A/duck/PhangNga/NIAH181/2004(H5N1) | Tai04p | Thailand |  | 2004-2-20 |
| AB450554 | A/quail/Phathumthani/NIAH2711/2004(H5N1) | Tai04q | Thailand |  | 2004-7-19 |
| AB450555 | A/chicken/Nonthaburi/NIAH2879/2004(H5N1) | Tai04o | Thailand |  | 2004-7-21 |
| AB450556 | A/chicken/NaraThiwat/NIAH1703/2004(H5N1) | Tai04m | Thailand |  | 2004-7-22 |
| AB450557 | A/chicken/Loei/NIAH2373/2004(H5N1) | Loei04 | Thailand |  | 2004-8-2 |
| AB450558 | A/chicken/Samutprakan/NIAH6604/2004(H5N1) | Tai04u | Thailand |  | 2004-8-5 |
| AB450560 | A/chicken/Nakhon Sawan/NIAH01503/2004(H5N1) | Tai04l | Thailand |  | 2004-10-22 |
| AB450562 | A/chicken/Suphanburi/NIAH7618/2004(H5N1) | Tai04w2 | Thailand |  | 2004-10-22 |
| AB450563 | A/duck/Angthong/NIAH8246/2004(H5N1) | Tai04e2 | Thailand |  | 2004-11-2 |
| AB450571 | A/open-bill stork/Thailand/VSMU-20-AYA/2004(H5N1) | Tai04x | Thailand |  | 2004-4-30 |
| AY553802 | A/little grebe/Thailand/Phichit-01/2004(H5N1) | Tai04c | Thailand |  | 2004-1-30 |
| AY651331 | A/Dk/Thailand/71.1/2004(H5N1) | Tai04b | Thailand |  | 2004-1-23 |
| AY770991 | A/ck/Ayutthaya/Thailand/CU-23/04 | Tai04d | Thailand |  | 2004 |
| DQ017277 | A/chicken/Sukhothai-2-01/2004(H5N1) | Tai04v | Thailand |  | 2004 |
| DQ017299 | A/chicken/Phetchabun-2-01/2004(H5N1) | Tai04r | Thailand |  | 2004 |
| DQ083573 | A/white peafowl/Bangkok/Thailand/CU-29/04(H5N1) | Tai04f | Thailand |  | 2004 |
| DQ083576 | A/chicken/Lopburi/Thailand/CU-38/04(H5N1) | Tai04k | Thailand |  | 2004 |
| EF467802 | A/ck/Thailand/2/04 | Tai04a | Thailand |  | 2004-1-21 |
| EF568923 | A/clouded leopard/Chonburi/AI-1216A/2004(H5N1) | Tai04g | Thailand |  | 2004 |
| EF582397 | A/duck/Kamphaengphet/NIAH6-2-0043/2004(H5N1) | Tai04i | Thailand |  | 2004-11-2 |
| FJ265565 | A/quail/Thailand/Phichit-01/2004(H5N1) | Tai04s | Thailand |  | 2004 |
| FJ265567 | A/pigeon/Thailand/Uttaradit-01/2004(H5N1) | Tai04t | Thailand |  | 2004 |
| EF619980 | A/turkey/Turkey/1/2005(H5N1) | Turkey05 | Turkey |  | 2005 |
| CY017051 | A/quail/VietNam/15/2005(H5N1) | Vn05n | Vietnam |  | 2005 |
| CY029511 | A/dk/Vietnam/1228/2005 | Vn05d | Vietnam |  | 2005-10-1 |
| CY029519 | A/dk/Vietnam/1231/2005 | Vn05e | Vietnam |  | 2005-11-1 |
| CY029527 | A/duck/Vietnam/1233/2005(H5N1) | Vn05f | Vietnam |  | 2005-6-27 |
| CY029543 | A/dk/Vietnam/1469/2005 | Vn05g | Vietnam |  | 2005-11-1 |
| CY029551 | A/dk/Vietnam/1771/2005 | Vn05h | Vietnam |  | 2005-10-1 |
| CY033185 | A/duck/Vietnam/NCVD08/2005(H5N1) | Vn05k | Vietnam |  | 2005 |
| DQ497705 | A/wild bird/Vietnam/434/2005(H5N1) | Vn05o | Vietnam |  | 2005 |
| DQ497717 | A/duck/Vietnam/543/2005(H5N1) | Vn05i | Vietnam |  | 2005 |
| EU124168 | A/Chicken/Vietnam/Long An 636/2005(H5N1) | Vn05c | Vietnam |  | 2005 |
| EU124169 | A/Chicken/Vietnam/Binh Duong477/2005(H5N1) | Vn05b | Vietnam |  | 2005 |
| EU124175 | A/Duck/Vietnam/Soc Trang 680C/2005(H5N1) | Vn05l | Vietnam |  | 2005 |
| EU124273 | A/duck/Vietnam/An Giang 680B/2005(H5N1) | Vn05j | Vietnam |  | 2005 |
| EU930876 | A/chicken/Vietnam/200/2005(H5N1) | Vn05a | Vietnam |  | 2005 |
| EU930980 | A/Muscovy duck/Vietnam/213/2005(H5N1) | Vn05m | Vietnam |  | 2005 |
| FJ877137 | A/mallard/AB/431/2006 | Outgroup |  |  | 2006 |
| EU016354 | A/duck/Switzerland/V487/2006(H5N1) | Switz06b | Switzerland |  | 2006 |
